# Soluble factors in COVID-19 mRNA vaccine-induced myocarditis causes cardiomyoblast hypertrophy and cell injury: a case report

**DOI:** 10.1186/s12985-023-02120-0

**Published:** 2023-09-03

**Authors:** Jose Gildardo Paredes-Vazquez, Nestor Rubio-Infante, Hector Lopez-de la Garza, Marion E. G. Brunck, Jaime Alberto Guajardo-Lozano, Martin R. Ramos, Eduardo Vazquez-Garza, Guillermo Torre-Amione, Gerardo Garcia-Rivas, Carlos Jerjes-Sanchez

**Affiliations:** 1 Tecnologico de Monterrey, Instituto de Cardiología y Medicina Vascular, Monterrey, Nuevo Leon Mexico; 2 Tecnologico de Monterrey, Institute for Obesity Research, Monterrey, Nuevo Leon 3000 CP 64710 Mexico

**Keywords:** COVID-19, Vaccine-induced myocarditis, Inflammation, COVID-19 mRNA vaccine

## Abstract

**Background:**

Inflammation affecting the heart and surrounding tissues is a clinical condition recently reported following COVID-19 mRNA vaccination. Assessing trends of these events related to immunization will improve vaccine safety surveillance and best practices for forthcoming vaccine campaigns. However, the causality is unknown, and the mechanisms associated with cardiac myocarditis are not understood.

**Case presentation:**

After the first dose, we reported an mRNA vaccine-induced perimyocarditis in a young patient with a history of recurrent myocardial inflammation episodes and progressive loss of cardiac performance. We tested this possible inflammatory cytokine-mediated cardiotoxicity after vaccination in the acute phase (ten days), and we found a significant elevation of MCP-1, IL-18, and IL-8 inflammatory mediators. Still, these cytokines decreased considerably at the recovery phase (42 days later). We used the cardiomyoblasts cell line to test the effect of serum on cell viability, observing that serum from the acute phase reduced the cell viability to 75%. We did not detect this toxicity in cells when we tested serum from the patient in the recovery phase. We also tested serum-induced hypertrophy, a phenomenon in myocarditis and heart failure. We found that acute phase-serum has hypertrophy effects, increasing 25% of the treated cardiac cells’ surface and significantly increasing B-type natriuretic peptide. However, we did not observe the hypertrophic effect in the recovery phase or sera from healthy controls.

**Conclusion:**

Our results opened the possibility of the inflammatory cytokines or serum soluble mediators as key factors for vaccine-associated myocarditis. In this regard, identifying anti-inflammatory molecules that reduce inflammatory cytokines could help avoid vaccine-induced myocardial inflammation.

Safe and effective vaccines for SARS-CoV-2 are critical to ending the pandemic [1]. The messenger RNA (mRNA) vaccines against COVID-19 have demonstrated unprecedented efficacy in preventing symptomatic infection and severe illness [2]. The mRNA technology includes a pre-fusion SARS-CoV-2 spike glycoprotein (S) antigen encoded in the mRNA and formulated in lipid nanoparticles, representing a novel vaccination technology with ongoing surveillance for potentially unrecognized side effects [3]. Two mRNA vaccines (BNT162b2, Pfizer-BioNTech; and mRNA-1273, Moderna) were the first SARS-CoV-2 vaccines authorized in the US [1]. Significant phase 3 trials for BNT162b2 and mRNA-1273 demonstrated that both were more than 94% effective with a low incidence of serious adverse events [1]. In addition, the Centers for Disease Control and Prevention recently reported rare cases of mRNA vaccine-induced myocardial inflammation [4] confirmed on cardiac imaging, serum cardiac biomarkers, antibody response, cytokine profile, and genetic findings [3, 5]. Characteristically, several distinct self-limited pericardial and myocardial inflammatory syndromes [4]. However, the causality is uncertain, and the mechanisms are unclear [3, 6]. After the first dose, we reported an mRNA vaccine-induced perimyocarditis in a young patient with a history of recurrent myocardial inflammation episodes and progressive loss of cardiac performance. We performed myocardium inflammatory characterization through cardiac magnetic resonance (CMR). We also analyzed the systemic inflammatory cytokines and the immune cell phenotype observed in vaccine-induced myocarditis compared with a healthy control group.

## Case Presentation

This case involves a 29-year-old male with a past medical history of three episodes of perimyocarditis during the last five years (the previous 21 months) characterized by a progressive decrease in the left ventricular global longitudinal strain (LVGLS) since the last event (Table 1).

**Table 1:** Clinical presentation, CMR findings, and treatment of recurrent myocarditis and pericarditis

Variables	February 2016	December 2017	May 2019	April 2021
**Signs and Symptoms**	Diarrhea, abdominal pain, fever, myalgias, joint pain, and sudden sharp chest pain	Fever, arthralgias, and stabbing chest pain	Recurrent stabbing chest pain (10/10) and myalgias	Abdominal pain, cramps, diarrhea, and recurrent severe pleuritic chest pain.
**Last of symptoms**	4	3	5	2
**ECG findings**	Extensive ST elevation (Fig. 1A)	Extensive ST-depression and T wave inversion V1-V2 (Fig. 1B)	Extensive ST-depression	Normal ECG. (Fig. 1C)
**CMR findings**	Mild LV dilation, LVEF 44%, LVGLS − 22.19%, anterolateral and anteroseptal myocardial edema, and anterolateral subepicardial LGE.	LVEF 60%, LVGLS − 20.07%, inferolateral and inferoseptal myocardial edema, same subepicardial LGE.	LVEF 58%, LVGLS − 17.03%, myocardial lateral wall edema, mid-wall lateral LV, and inferior RV free wall LGE.	LVEF 57%, LVGLS − 12.61%, reduced reservoir left atrial strain (-22.25%) and conduit left atrial strain (-14.0%), pericardial LGE areas (anterior, inferior, and lateral myocardial walls).
**US-CRP (mg/dL)**	8.48	8.14	4.42	5.51
**CD4+/CD8 + T Cell Ratio**	-----	-----	-----	1.02
**scTn > 0.05 ng/mL**	6.15	1.06	-----	-----
**hs-cTnI (ng/L)**	-----	-----	11416.8	805.1
**BNP (pg/mL)**	63.8	16.1	< 10	30.1
**D-dimer (ng/mL)**	192	ND	ND	ND
**Treatment**	Aspirin 100 mg PO QD Carvedilol 6.25 mg PO BID Candesartan 8 mg PO QD	Colchicine 0.5 mg PO BID Ibuprofen 800 mg PO TID Morphine Sulphate 2.5 mg IV	Colchicine 0.5 mg PO QD Indomethacin 50 mg PO TID Metoprolol succinate 95 mg ¼ QD Morphine Sulphate 5 mg IV	Colchicine 0.5 mg PO BID Ibuprofen 800 mg PO TID

LVEF: left ventricular ejection fraction; LVGLS: left ventricular global longitudinal strain; LGE: late gadolinium enhancement; USCRP: ultra-sensitive C-reactive protein; scTn: standard cardiac troponin; hs-c-TnI: high sensitive cardiac troponin I; BNP: B-type natriuretic peptide

**Fig. 1:**
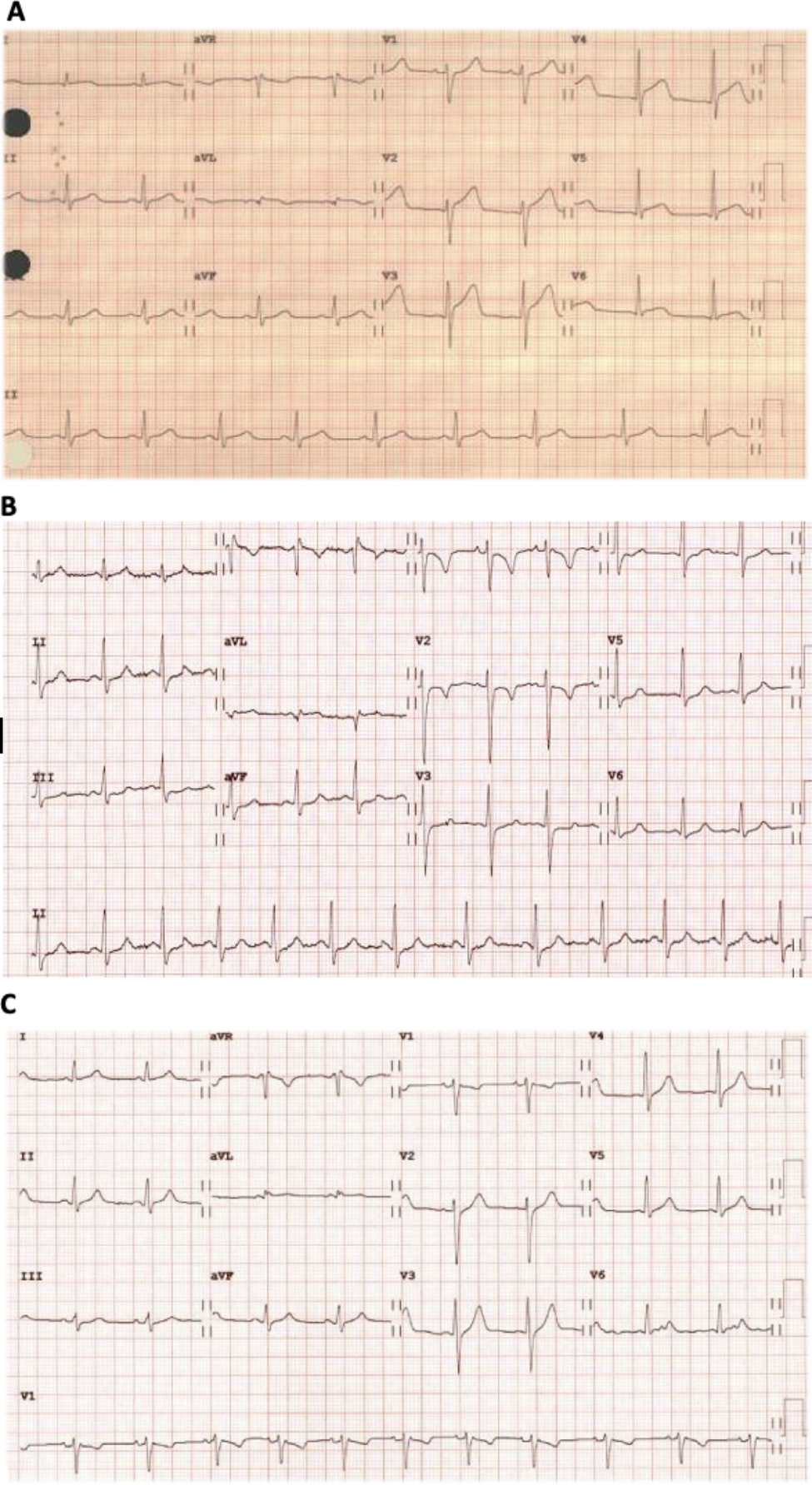
Myocarditis and pericarditis ECG findings A: February 2016, shows anterior ST elevation. B: December 2017, shows anterior ST depression and T wave inversion. C: April 2021, normal ECG.

Ten days before, he received the first dose of the anti-SARS-CoV-2 vaccine (Pfizer BioNTech) without immediate side effects. The patient arrived at the ER on Apr 4, 2021, with a 48-hour history of mild abdominal pain, cramps, and diarrhea. He presented with severe pericardial pain resembling previous perimyocarditis episodes five hours before arrival. For the last 21 months, the current medication was colchicine 0.5 mg OD [7, 8] and metoprolol succinate 95 mg OD. Vital signs showed blood pressure 120/60 mmHg, heart rate 88 bpm, respiratory rate 14 rpm, temperature 36.8 ºC, and O2 saturation at room air 97%. The physical examination was unremarkable. The initial ECG revealed sinus arrhythmia, and the chest X-ray was normal upon admission. Figure 1 shows myocarditis and pericarditis ECG findings.

**Fig. 2:**
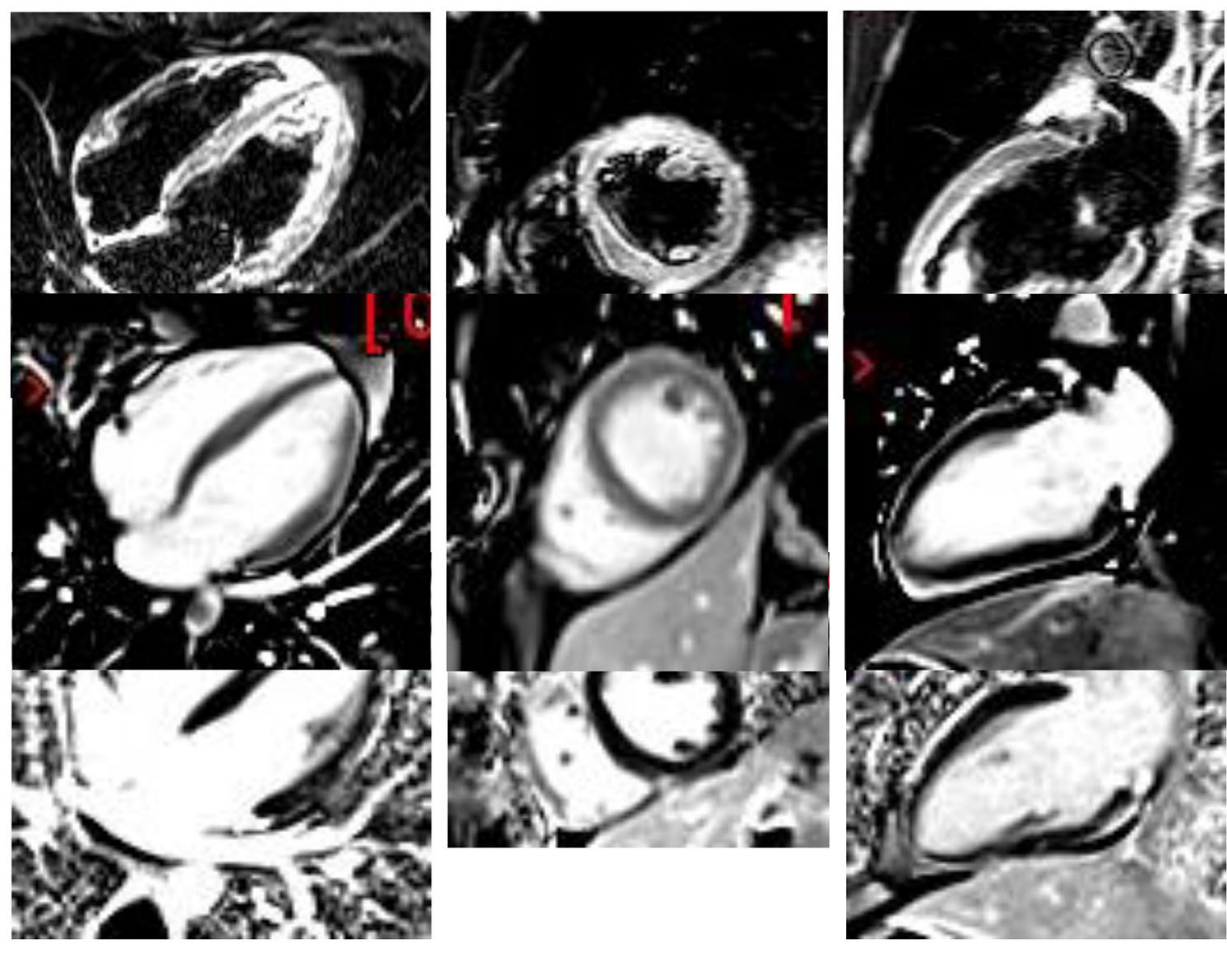
CMR during myocarditis episode TIRM sequence with myocardial edema in anterior, lateral, and inferior wall. Second line: EGE without enhancement. Third line: LGE with an enhancement of the pericardium in anterior, lateral, and inferior wall

Initial laboratory and inflammatory cytokine findings demonstrated ultra-sensitive C-reactive protein (CRP) (5.51 mg/dL) and high-sensitivity cardiac troponin I (hs-cTnI) abnormal measurements (805.1 ng/L) with normal B-type natriuretic peptide (BNP) (30.1 pg/mL), and high levels of IL-8, IL-18, and MCP-1 (Table 2). The nasopharyngeal RT-PCR SARS-CoV-2 was negative. The patient was admitted, and we started an anti-inflammatory treatment with oral colchicine [7, 8] and ibuprofen. CMR with late gadolinium enhancement (LGE) showed normal right (RVEF 54.2%) and left (LVEF 57%) ventricular systolic function with abnormal LVGLS (-12.61%) and a normal right ventricle global longitudinal strain (RVGLS) (-21.0%). We also detected a reduced reservoir left atrial strain (-22.25%) and conduit left atrial strain (-14.0%) [9], with mild mitral regurgitation (regurgitation fraction 10%) without myocardial early gadolinium enhancement (EGE) images (Fig. 2). In addition, we identified multiple pericardial LGE areas (anterior, inferior, and lateral myocardial walls) (Fig. 2 ).

**Fig. 3:**
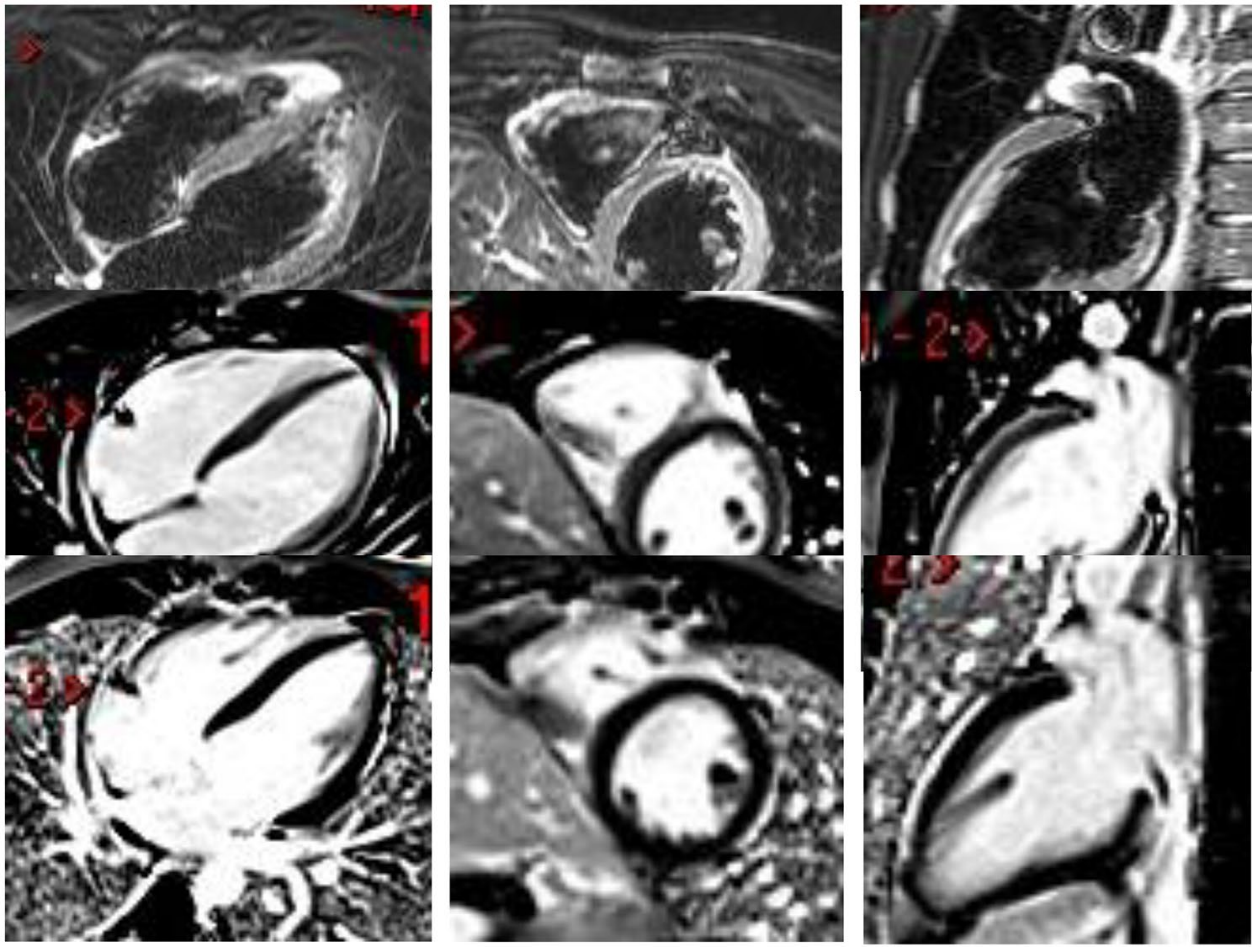
Cardiac magnetic resonance follow-up First line: TIRM sequence diminishing of myocardial edema. Second line: EGE without enhancement. Third-line LGE with a remaining enhancement of the pericardium in the anterior, lateral, and inferior wall.

The in-hospital stay was uneventful, and we discharged the patient with oral colchicine, ibuprofen, and metoprolol succinate. The patient was asymptomatic and in functional class, I, significantly improving CMR findings in the follow-up (Fig. 3).

He received the second anti-SARS-CoV-2 vaccine four weeks after the perimyocarditis episode without any side effects. On day 42, since a hospital admission, the patient returned to the hospital for a routine observation (recovery phase).

## Methods

### Human samples

Healthy human samples were collected from control patients enrolled in the protocol of pulmonary arterial hypertension approved by the Research and Ethical Committee of Escuela de Medicina y Ciencias de la Salud. Tecnologico de Monterrey on May 17, 2021, where they conceit for future use of their samples for research purposes.

### FACS analysis

Peripheral blood mononuclear cells were used to determine T cells (CD3+, CD4+, CD8+) NK cells (CD56+), Monocytes (CD14+, CD16+), and B cells (CD19+). In brief, cells were resuspended in mouse anti-human CD3-FITC (Biolegend® cat. 300,406), mouse anti-human CD4-PE (Biolegend® cat. 357,403), mouse anti-human CD8-APC (Biolegend® cat. 344,721), mouse anti-human CD56-PE (Biolegend® cat. 304,606), mouse anti-human CD16-PeCy7 (Biolegend® cat. 302,016), mouse anti-human CD20-PerCP Cy5.5 (Biolegend® cat. 302,229) or mouse anti-human CD14-APC (Biolegend® cat. 301,807), in a final 150µL staining volume with PBS + 2% FBS per 1 × 10^6^ cells. Samples were incubated for 30 min on ice in the dark, then washed twice and resuspended in 150 µL PBS/2% FBS prior to analysis; approximately 1 × 10^6^ mononuclear cells were kept as unstained control. Samples were acquired on a BD® FACSCanto II flow cytometer with a 488-nm, and 633-nm lasers and operated through the BD® FACSDiva software v.8. Cytometer was calibrated before all acquisition using CS&T beads (BD® cat. 642,412) according to manufacturer’s instructions. We use flow cytometry data analyzed FlowJo software v.10 (Treestar LLC). Automatic compensation was performed before analysis, with a compensation matrix generated at each acquisition.

### Cytokine measurement

Cytokines plasmatic levels were measured by flow cytometry using LEGENDplex™ Human Inflammation Panel 1 kit (13-plex) (Biolegend: Legendplex, San Diego, CA, USA) as per manufacturer description but with two additional dilution points on the calibration curve. FACS-Canto II equipment was used for the flow cytometer (Becton Dickinson, Franklin Lakes, NJ, USA).

### Cellular viability

Cardiomyoblasts (1000 cells/well) were seeded with Dulbecco´s Modified Eagle Medium (DMEM) supplemented with 10% of Fetal Bovine Serum (FBS); after 24 h medium was changed to 1% of FBS and incubated for 48 h with 5% of plasma derived from a healthy human control or from the myocarditis patient (during acute and recovery phases). Cell viability was determined by an AlamarBlue assay. For statistical analysis, an ANOVA was calculated considering p < 0.05 and p < 0.01.

In vitro hypertrophy assay.

To evaluate cellular hypertrophy after serum stimulation, cardiomyoblast were seeded in 6-well plates (2000 cells/well) over round coverslips. Cells were then treated as specified in the cell viability assay and after 48 h of incubation with a serum derived from a patient or from a healthy subject, cells were stained using calcein-AM (5 µM, Invitrogen, C34852) incubating for 30 min at 37 °C. Afterward, coverslips were mounted in a superfusion chamber, and added the nuclear-stain Draq5 (20µM, Thermo Scientific, 62,251) was before analysis. Images were acquired using a Leica TCS SP5 confocal microscope equipped with a D-apochromatic 40X, 1.2 NA, oil objective (Leica Microsystems, Wetzlar, Germany), and analyzed using ImageJ software (http://imagej.nih.gov/ij/, NIH, Bethesda, MD, USA) to quantify cell’s surface.

### qPCR gene expression

RNA was purified by the Trizol method and cDNA was synthesized using the BRAND, CAT kit. HPRT was used as a control gene. For statistical analysis, an ANOVA was performed considering p < 0.05 and p < 0.01.

### Neutralization assay

We used the pseudovirus‑based platform published by Cruz-Cárdenas et al. 2022. On the day of the assay, sera were serially diluted by 7-folds, spanning 1:5 to 1:9860, and 100 µL of each dilution was incubated for 1 h at 37 °C and 5% CO2, with 15 pg of SARS-CoV-2 viral particle (VP) by duplicate in a 96-well plate. Post-incubation, 25,000 Vero cells were seeded to each well and the plate was incubated for 24 h at 37 °C and 5% CO2. As a positive control for transduction, SARS-CoV-2 VP was incubated with Vero cells. After 24 h, neutralization was measured and reported as % of inhibition of transduction [10].

### Experimental results

#### Abnormal immune cells´ subsets in vaccine-induced myocarditis

We observed an irregular distribution of immune subsets in PBMC derived from the vaccine-induced myocarditis patient (acute phase) with higher levels of T CD8 + cells, and a diminished of CD4 + cells in the patient compared with a control group; the ratio of CD4+/CD8 + T cells (1.02) was reduced by 49.08% compared with the control group, possibly explained by the CD8 + increase. NK cells were also increased by five-fold, and non-classic monocytes were increased by 1.67-fold compared with control subjects (Table 2). The CD4+/CD8 + ratio continued to diminish maybe by the regular immunosuppressor therapy with cyclosporine.

Increased pro-inflammatory cytokines and overproduction of SARS-CoV-2 neutralized antibodies in vaccine-induced myocarditis. The inflammatory cytokines profile was characterized in the vaccine-induced myocarditis at the acute phase. We found a substantial elevation of MCP-1, IL-8, and IL-18 inflammatory mediators. We also observed an increase in IL-10 and IL-33 (Table 2).

**Table 2:** Laboratory, biomarkers and cytokine pro-inflammatory panel

Blood count	Reference range	Acute phase	Recovery phase
Hemoglobin (gr/dL)	13.2–18.0	16.4	-
Hematocrit (%)	38.4–52.4	46.7	-
Platelet count (x 10^3/uL)	150.0-420.0	227	-
White-cell count (x 10^3/uL)	4.5–11.0	6.7	-
Absolute neutrophil count (x 10^3/uL)	2.5-7.0	4.1	-
Absolute lymphocyte count (x 10^3/uL)	1.0–4.0	1.3	-
Absolute monocyte count (x 10^3/uL)	0.2–1.2	1.0	-
Comprehensive Metabolic Panel			
Creatinine (mg/dL)	0.7–1.3	1.1	-
Blood urea nitrogen (mg/dl)	8.9–25.7	11.5	-
Lactate dehydrogenase (U/L)	125.0-243.0	254	-
Inflammatory biomarkers			
C-reactive protein (mg/dL)	0.0-0.5	5.51	-
Erythrocyte sedimentation rate (mm/hr)	1–20	3.0	-
Procalcitonin (ng/mL)	< 0.5	< 0.05	-
Cardiac biomarkers			
hs-c-TnI (ng/L)	0.0-34.2	805.1	-
BNP (pg/mL)	< 100	30.1	-
D-Dimer (ng/mL)	< 243	54.0	-
Pro-inflammatory cytokine	Control group*		
IL-1β	0.5 ± 1.3	< 11.88	< 11.88
IFN-α2	41.6 ± 106.5	< 9.61	< 9.61
IFN-γ	1.7 ± 2.7	< 17.33	34.43
TNF-α	1.7 ± 3	< 20.06	< 20.06
MCP-1	0.1 ± 70.5	1220.2 ↑	764.4 ↑
IL-6	0.7 ± 0.9	< 1.37	2.61 ↑
IL-8	1.5 ± 2.3	47.23 ↑	< 5.37
IL-10	1.0 ± 1.4	24.74 ↑	< 0.69
IL-12p70	0.6 ± 0.7	< 0.06	< 0.06
IL-17 A	6.3 ± 20.2	< 113.22	< 113.22
IL-18	169.6 ± 94.2	2177.1 ↑	1516.7 ↑
IL-23	4.3 ± 8.8	5.30	5.30
IL-33	2.5 ± 3.4	4.6 ↑	2.6

On day 42, the inflammatory and cardiac biomarkers findings. Also, pro-inflammatory cytokine determination in the acute phase at day seven and the recovery phase of myocarditis at day 42 after vaccination. * Control: ten healthy subjects.

The patient returned to the hospital for a routine observation, PBMC, plasma, and serum were obtained at this visit (recovery phase), Interestingly at the recovery phase, the levels of MCP-1, IL-8, IL-10, IL-18, and IL-33 were diminished considerably compared with the acute phase but were still higher than the control levels (Table 2). We also found elevated SARS-CoV-2 viral neutralization titer 50 (VNT50) of 1:273 after the first vaccine dose at the moment of the ER arrival.

**Fig. 4:**
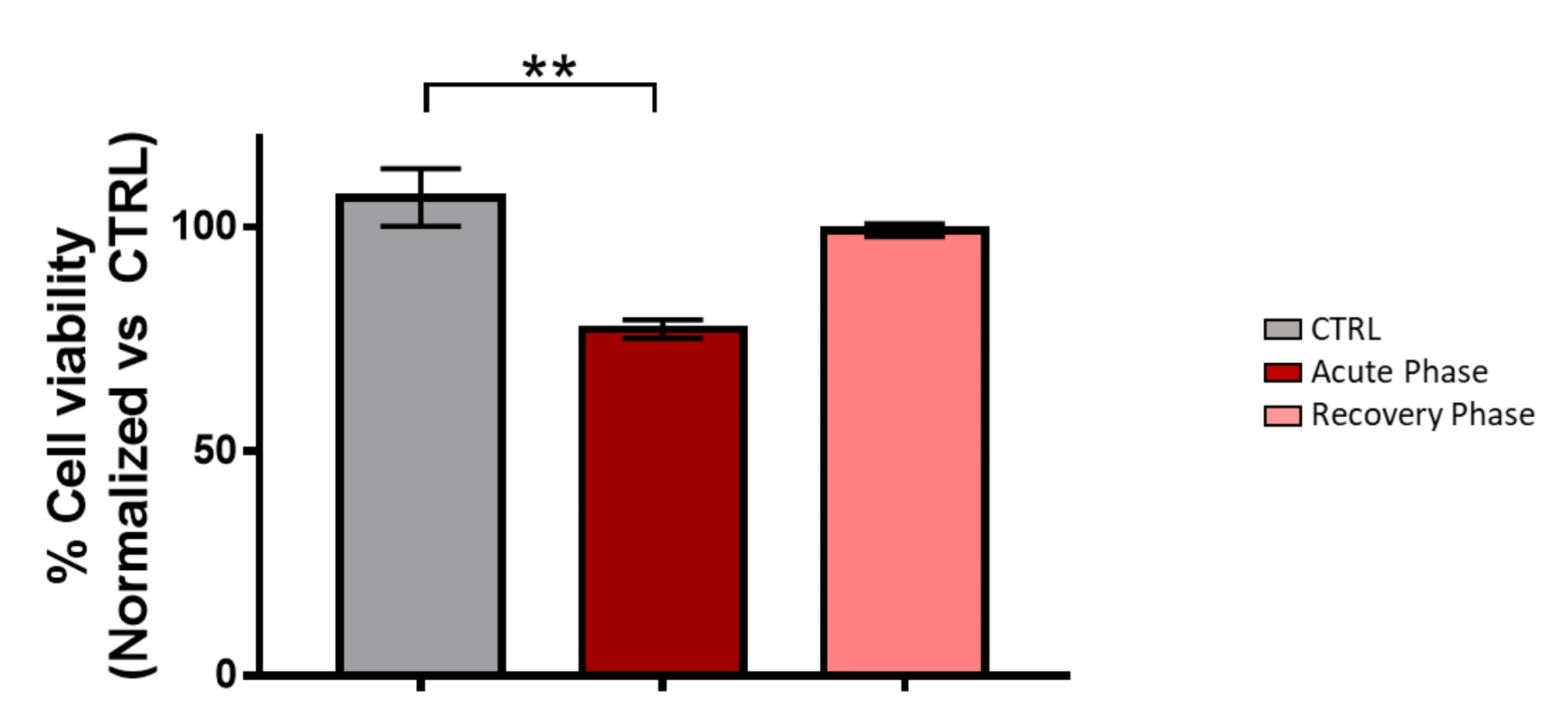
Cellular viability of sera-treated groups. Cells were seed in DMEM supplemented with 10% FBS, after 24 h medium was changed to 1% FBS, and 5% of plasma was added from a healthy human control or myocarditis patient (during acute and recovery phases) for 48 h. Cell viability was determined by an Alamar blue assay. For statistical analysis, an ANOVA was calculated considering p < 0.01 **

**Fig. 5:**
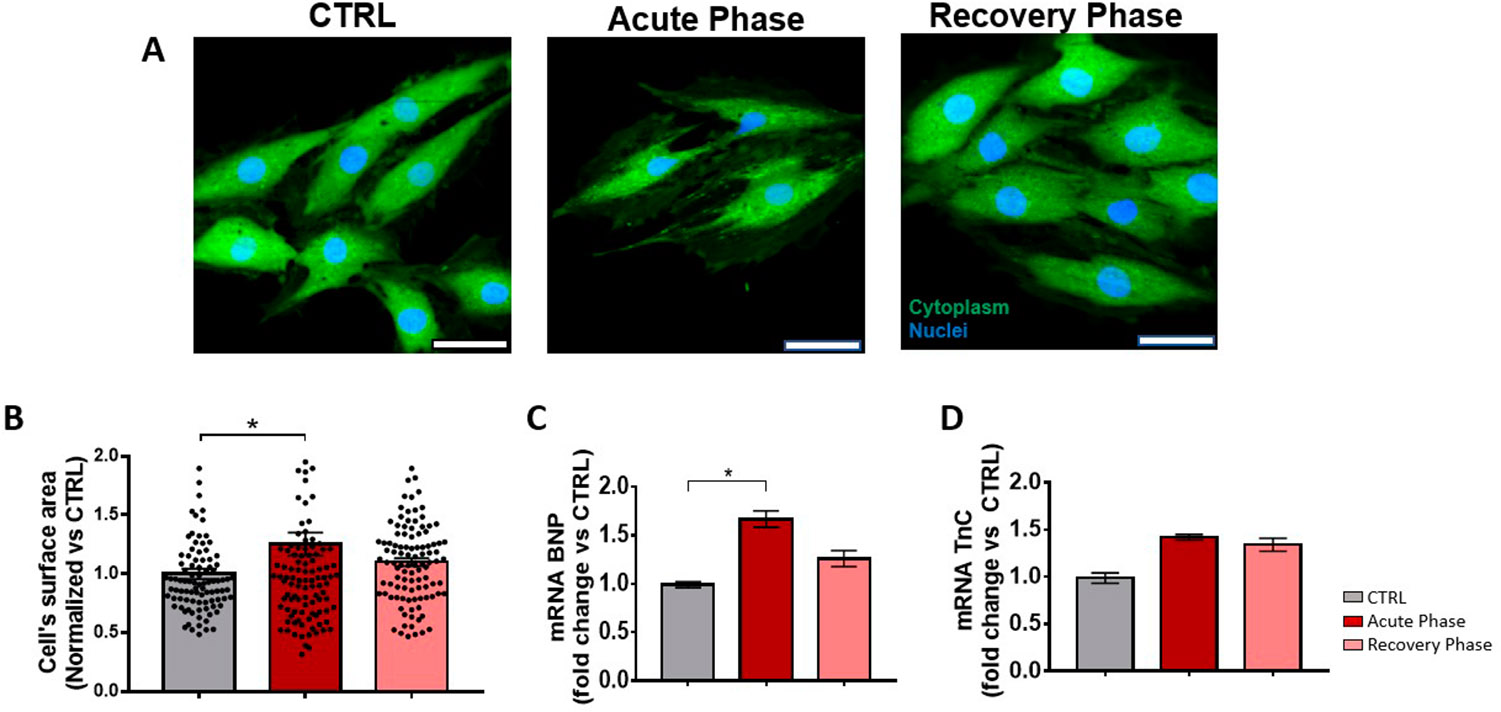
Serum from a patient with Vaccine-induced myocarditis provokes hypertrophy, increasing BNP expression. (**A**) Hypertrophy assay, representative confocal images of treatments are shown (Green calcein-stained cytoplasm and Blue Draq5 stained nuclei) Hypertrophy assay, representative confocal images of treatments are shown (Green calcein-stained cytoplasm and Blue Draq5 stained nuclei). **(B)** Cell-surface quantification in treated groups, CTRL, (Gray bars), Acute recovery (Red bars), recovery (Pink bars). **C and D.** mRNA expression of stress and hypertrophic markers BNP and TnC, respectively. RNA was purified by the Trizol method and converted to cDNA to perform a qPCR. HPRT was used as a control gene. For statistical analysis, an ANOVA was calculated considering mRNA expression of stress and hypertrophic markers BNP and TnC, respectively. RNA was purified by the Trizol method and converted to cDNA to perform a qPCR. HPRT was used as a control gene. For statistical analysis, an ANOVA was calculated considering p < 0.05 * and p < 0.01 **. We also determined cardiac stress and injury markers after cells’ treatment with control serum, acute-phase serum, and recoveryphase serum. Only the serum in the acute phase increased BNP, a cardiac marker of hemodynamic stress and ventricular dysfunction (Fig. 5-**C**). We did not observe the effects of sera treatments on the hs-cTnI expression (Fig. 5-**D**)

Serum derived from vaccine-induced myocarditis patients decreases cell viability and cardiomyoblast hypertrophy. We used cardiomyoblast in culture to evaluate the effect of serum over cell viability (Fig. 4); serum from the acute phase reduced the viability to approximately 75%; this diminution was neither observed with the serum derived from the patient in the recovery phase (where the clinical parameters had been improved) nor with the serum from a healthy control. We also assessed serum-induced hypertrophy, a phenomenon presented in cardiac pathologies such as myocarditis, and heart failure. We used the same samples and conditions from the viability assay. We found that acute phase-serum had hypertrophic effects, increasing the surface of the treated cardiac cells by 25%. However, the hypertrophic effect was not observed in the patient’s recovery phase or healthy control sera (Figs. 5-A and 5-B).

We also determined cardiac stress and injury markers after cells’ treatment with control serum, acute-phase serum, and recovery-phase serum. Only the serum in the acute phase increased BNP, a cardiac marker of hemodynamic stress and ventricular dysfunction (Fig. 4-C). We did not observe the effects of sera treatments on the hs-cTnI expression (Fig. 4-D).

## Discussion

The key findings, in this case, are as follows: (a) the unusual three previous recurrent episodes of perimyocarditis confirmed by CMR and the progressive decrease in the LVGLS since the last event (Table 1); (b) the abnormal LVGLS (-12.61%), abnormal reservoir left atrial strain (-22.25%), and abnormal conduit left atrial strain (-14.0%) [9, 11], suggesting an early left ventricular dysfunction stage; (c) the myocardial inflammation after ten days of the first dose of the COVID-19 mRNA vaccine; (d) the systemic inflammatory cytokine increased in the acute phase; (e) the elevated SARS-CoV-2 viral neutralization titer 50 (VNT50) of 1:273 after the first dose, which is very similar to the reported VNT50 after the second dose of the Pfizer vaccine (1:312 after seven days and 1:169 after 21 days) [12]; and (f) the viral panel and the clinical data which discard a virus as a causal agent.

Vaccination is a well-established part of preventive and public health medicine but is not risk-free [3]. For example, historically, rare vaccine-induced myocardial inflammation cases were reported following a live smallpox vaccine (incidence of 2.16–7.8 per 100,000 vaccines), with reports occurring up to 30 days post-vaccination [2]. The Covid-19 mRNA vaccine-induced myocardial inflammation is mainly among males aged 12 to 39 years, with about 4.8 cases per one million and ≈ 12.6 cases per million of second doses [4, 13]. Although myocarditis and pericarditis after vaccination for COVID-19 are two distinct self-limited syndromes [4], myocarditis develops rapidly in younger patients, mainly after the second dose of vaccination [4] pericarditis, affects older patients later, after the first or second dose [4]. Our patient had a proximate vaccine dose, onset symptoms period, clinical presentation, recovery, and CMR findings (Fig. 1) consistent with an mRNA vaccine-induced perimyocarditis.

The underlying mechanisms of COVID-19 mRNA vaccine-induced myocarditis are not precise. However, some hypotheses suggest that the lymphocytic infiltration results in an immune-mediated myocardial injury [3]; molecular mimicry between the spike protein of SARS-CoV-2 and self-antigens could be possible; COVID-19 mRNA vaccine could enhance preexisting dysregulated immune pathways or inflammatory cytokine production [13]. Here, we observed a potential inflammatory cytokine-mediated cardiotoxicity in the acute phase with a considerable decrease in the recovery phase, but still higher than the control group (10 pre-pandemic PBMC of healthy subjects). Inflammatory cytokines and chemokines are key soluble factors in cardiac diseases such as heart failure and myocarditis. We have demonstrated that inflammatory cytokines such as TNF-α, IL-6, and IL-1β are soluble mediators linked with ventricular arrhythmias and contractile dysfunction in a rat model of metabolic syndrome [14]. Experimental evidence shows that IL-1β induces cardiac fibrosis and hypertrophy and depresses cardiac function. Here, we found an essential increase of the chemokine MCP-1 in the acute phase of vaccine-induced perimyocarditis, MCP-1 increases the migration of monocytes, and data from animal models and in vitro experiments suggest that MCP-1 can promote cardiac fibrosis [15, 16]. The recombinant expression of MCP-1 in cardiac tissue induces myocarditis, cardiac hypertrophy, and dilation. In addition, there is evidence that MCP-1 is critical in the induction of the experimental autoimmune myocarditis model, and its inhibition significantly reduced disease severity [17]. We found the elevation of IL-18, a cytokine, is involved in animal models of myocardial infarction with pressure overload effects. The increased risk of cardiovascular disease correlates with high IL-18 levels [18]. We proposed that the cardiac injury triggers the inflammatory cascades by inducing IL-18 and chemokines such as MCP-1 and IL-8 (a critical regulator of neutrophil influx and activation in inflammatory processes [19]. The role of neutrophil extracellular traps in COVID-19 vaccine-induced myocarditis is unclear [20]. Also, the allopurinol effect on the oxidative stress index and endothelial dysfunction and direct cytopathic impact or indirectly through the propagation of pro-inflammatory cytokines inducing endothelial dysfunction and oxidative stress remain an unmet question [21]. The abnormal immune cell subsets, the increased inflammatory cytokines, and the augmented CRP levels show the patient’s inflammatory state after ten days of getting the COVID-19 mRNA COVID-19 vaccine, which may trigger cardiac injury and dysfunction.

COVID-19 vaccination’s side effects are usually minor (pain, swelling, redness at the injection site), systemic (fatigue, headache, muscle pain, chills, and fever), transient [22], and more common in younger males. They also occur more often after the second dose [22]. Our case’s principal findings were the abnormalities in the left atrial reservoir, conduit, and LVGLS as the earliest signs of myocardial dysfunction in the setting of preserved ejection fraction myocarditis (LVEF 57%), making the diagnosis of left ventricular dysfunction unlikely based on basic imaging parameters [9, 11]. Despite performing extensive viral PCR panels of respiratory, gastrointestinal, and rheumatologic systems, we could not locate a specific virus for previous acute events. We did not perform a myocardial biopsy because of the CMR findings and the clinical course, so we considered a possible viral etiology, the most frequent perimyocarditis cause. The contribution of this case report to the previous evidence is an unexpected myocarditis development after the first dose (most vaccine-associated myocarditis is seen after the second dose) [23]. Also, the significance of these findings is proof of concept that the cytokines/inflammatory factors/serum components may be the direct mediators of vaccine-associated myocarditis. Further, we opened the possibility of the inflammatory cytokine or serum soluble mediators as key factors for vaccine-associated myocarditis. In this regard, identifying anti-inflammatory compounds that reduce inflammatory cytokines could help to avoid vaccine-induced myocardial inflammation.

## Data Availability

Not applicable.
